# Significance of Exhaled Breath Test in Clinical Diagnosis: A Special Focus on the Detection of Diabetes Mellitus

**DOI:** 10.1007/s40846-016-0164-6

**Published:** 2016-10-11

**Authors:** Souvik Das, Saurabh Pal, Madhuchhanda Mitra

**Affiliations:** 1 Department of Biomedical Engineering, JIS College of Engineering, Kalyani, West Bengal 741235 India; 2 Department of Applied Physics, University of Calcutta, Kolkata, West Bengal 700009 India

**Keywords:** Breath analysis, Volatile organic compound, Disease diagnosis, Noninvasive method, Breath biomarkers, Diabetes mellitus

## Abstract

Analysis of volatile organic compounds (VOCs) emanating from human exhaled breath can provide deep insight into the status of various biochemical processes in the human body. VOCs can serve as potential biomarkers of physiological and pathophysiological conditions related to several diseases. Breath VOC analysis, a noninvasive and quick biomonitoring approach, also has potential for the early detection and progress monitoring of several diseases. This paper gives an overview of the major VOCs present in human exhaled breath, possible biochemical pathways of breath VOC generation, diagnostic importance of their analysis, and analytical techniques used in the breath test. Breath analysis relating to diabetes mellitus and its characteristic breath biomarkers is focused on. Finally, some challenges and limitations of the breath test are discussed.

## Introduction

Bioinformation obtained from volatile organic compounds (VOCs) in the exhaled breath of humans can aid the early diagnosis of several diseases and can be used to decide relevant medical therapies. The analysis of exhaled breath and associated VOCs has gained a considerable scientific, clinical, and research attention due to its potential in enabling the noninvasive observation of the biochemical processes of the human body [1–3]. The first initiatives of breath analysis for determining the physiological state of humans originated during the time of Hippocrates (460-370 BC), when the ancient Greek physicians realized that some diseases could be diagnosed from the characteristic odor of patients’ breath and knew that the human breath might provide sound information on health conditions [1–6]. In the period 1782–1783, Lavoisier for the first time analyzed the breath CO_2_ of Guinea pigs and showed that the gas is a product of combustion in the body [1, 5]. Practically, it is not difficult for a skilled technician to recognize the characteristic ‘fruity smell’ of acetone, ‘musty and fishy smell’, ‘urine-like smell’, and ‘putrid smell’ in the breath of patients with diabetes, advanced liver disease, kidney failure, and lung abscess, respectively [1]. The analysis of VOCs present in exhaled breath can thus provide valuable information about the subjects’ physiological and pathophysiological conditions. Such compounds can be useful indicators and potential biomarkers of various diseases and metabolic activities, facilitating disease diagnosis. It should be noted that biological monitoring is generally based on the analysis of blood. However, this involves an invasive and time-consuming technique, which is often unacceptable concerning patient care system. This invasive technique also needs skilled medical staff. Breath analysis is thus a very attractive alternative as it is a noninvasive and quick method that allows repeated sampling [1, 7].

A plethora of studies have reported the significance of analyzing VOCs in human breath [8–10]. In 1938, the ‘drunkometer’ was introduced as a roadside test of breath-alcohol concentration [7, 11]. In the 1970s, Pauling et al. [12] used gas chromatography (GC) to detect more than 200 VOCs in human breath. Their contribution gave new insight into the human body and directed research in the analysis of VOCs for clinical diagnosis and therapeutic monitoring [2, 13, 14]. Since then, many studies have been carried out in this field. In 1997, Phillips [15] estimated 1259 VOCs from 20 normal healthy subjects using gas chromatography–mass spectrometry (GC/MS). In 1999, using GC/MS, Phillips et al. described 3481 VOCs in the breath gas of healthy controls, with an average number of about 200 VOCs detectable in an individual’ breath gas [16]. Recently, 1765 VOCs of healthy humans have been published [5, 17].

Compounds in exhaled breath gas are generally classified as [3]:

1. Inorganic compounds, such as nitric oxide, oxygen, and carbon dioxide [18].
2. Exhaled breath condensate, which includes cytokines, hydrogen peroxide, isoprostanes, and leukotrienes [17].
3. VOCs, such as ethane, pentane, aldehydes, and isoprene [2, 18, 19].

In the context of the diagnosis of human diseases, a number of volatile biomarkers in exhaled breath have been reported [17, 18, 20–24]. It is essential to determine such biomarkers in order to monitor metabolic or any pathologic processes in the body. Expired breath VOCs that are commonly used for diagnostic purposes include oxygen-containing compounds (e.g., ethanol, methanol, 2-propanol, acetone, and acetaldehyde), hydrocarbons (e.g., ethane, pentane, and isoprene), sulfur-containing compounds (e.g., methyl mercaptanes, ethyl mercaptanes, and dimethyl sulfide), and nitrogen-containing substances (e.g., dimethylamine, trimethylamine, and ammonia) [2]. Acetone, isoprene, ethanol, methanol, other alcohols and alkanes have been reported to be the major identified VOCs [3, 25]. A few of the above-mentioned types of breath VOC along with their possible biochemical pathways in the human body are discussed below.

## Origin and Biochemical Pathways of Major VOCs in Human Exhaled Breath

### Oxygen-Containing Compounds

**Fig. 1:**
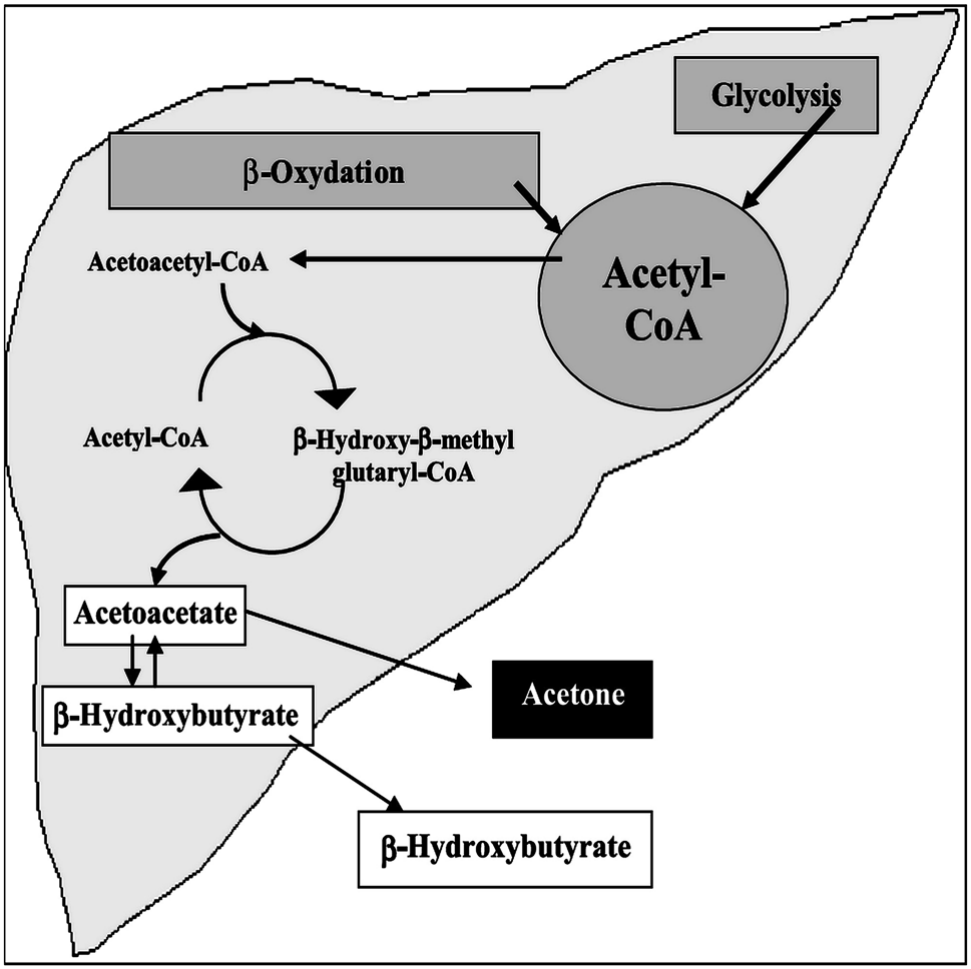
Generation pathway of acetone in liver by hepatocytes [2]

Acetone is one of the major abundant VOCs in human breath [2, 26]. It should be emphasized here that acetone production in animals is believed to originate from two pathways: the decarboxylation of acetoacetate [2, 26–29] and the dehydrogenation of isopropanol or 2-propanol [27–30]. The first pathway, the major source of acetone production in mammals, arises from either lipolysis (lipid peroxidation) or amino acid degradation [2, 26–29]. Acetoacetate decarboxylation may occur both in an enzyme-catalyzed manner and non-enzymatically. Studies on various rat tissues (e.g., kidney, liver, brain, placenta, and plasma) and human plasma [27, 29] have established the presence of enzymatic activity. Figure 1 illustrates the generation of acetone generated by hepatocytes via the decarboxylation of excess acetyl–coenzyme A (acetyl–CoA). The second metabolic pathway includes the dehydrogenation of 2-propanol (by liver alcohol dehydrogenase, ADH) to acetone. This acetone is released mostly via urine and exhaled breath from the body. Acetone may also be further metabolized to acetate, formate, and carbon dioxide [30]. Acetone, a natural metabolic intermediate of lipolysis, is as a potential biomarker for monitoring the ketotic state of diabetic and fasting individuals, assessing fat loss, and measuring glucose levels [26].

In the human body, the origins of ethanol and methanol are not well understood [31–33]. However, these VOCs are believed to originate from microbial fermentation of the carbohydrates in the gastro-intestinal tract [31, 34–36]. Intestinal bacterial flora is regarded as an important source of breath ethanol [37] and methanol [38]. It has been proposed that methanol derives from S-adenosylmethionine in the pituitary or from the breakdown of ethanol by intestinal flora [32]. Usually, the breath ethanol concentrations in human subjects are much lower than those found in human breath after the intake of alcohol. It has also been postulated that an enzyme-mediated reduction of acetone forms 2-propanol in the body, implying that its concentration in humans remain lower than acetone concentrations [38].

Researchers also claim ethanol and methanol to be mostly of microbial or exogenous origin [35]. Sources of exogenous methanol in the healthy human body include fruits, vegetables, and alcoholic beverages [35]. Methanol levels in breath may be influenced by intake of fruit-derived pectin [36]. In a recent study on C57BL*/*6J mice revealed that methanol source strengths are barely affected by body mass. Rather, they are influenced by the diet matrix and a low-fat diet [36]. Regardless of the source, low levels of methanol are always maintained by the human physiological system [35].

Acetaldehyde originates as the first product, primarily in the liver during the metabolism of ethanol. Here, ethanol is oxidized by ADH. The enzyme aldehyde dehydrogenase converts the produced acetaldehyde to acetate [39]. Breath acetaldehyde concentration is lower than ethanol concentration [2].

### Hydrocarbons

#### Saturated Hydrocarbons

The major fractions of exhaled ethane and pentane are of systemic origin [2, 40]. These compounds are thought to originate from lipid peroxidation, a chain reaction that starts when an allylic hydrogen atom is eliminated by reactive oxygen species (ROS). The resulting radical is conjugated, peroxidized by oxygen, and undergoes further chemical reactions. The generation of ROS is described as the destructive aspect of oxidative stress [41]. In oxidative stress, cells are damaged as a result of a chemical reaction with oxidative agents (e.g., the superoxide anion or hydroxyl radical) [6, 42]. ROS bear free radicals and peroxides. These species may undergo further chemical changes and turn into more aggressive radical agents that can potentialy cause extensive cellular damage.

**Fig. 2:**
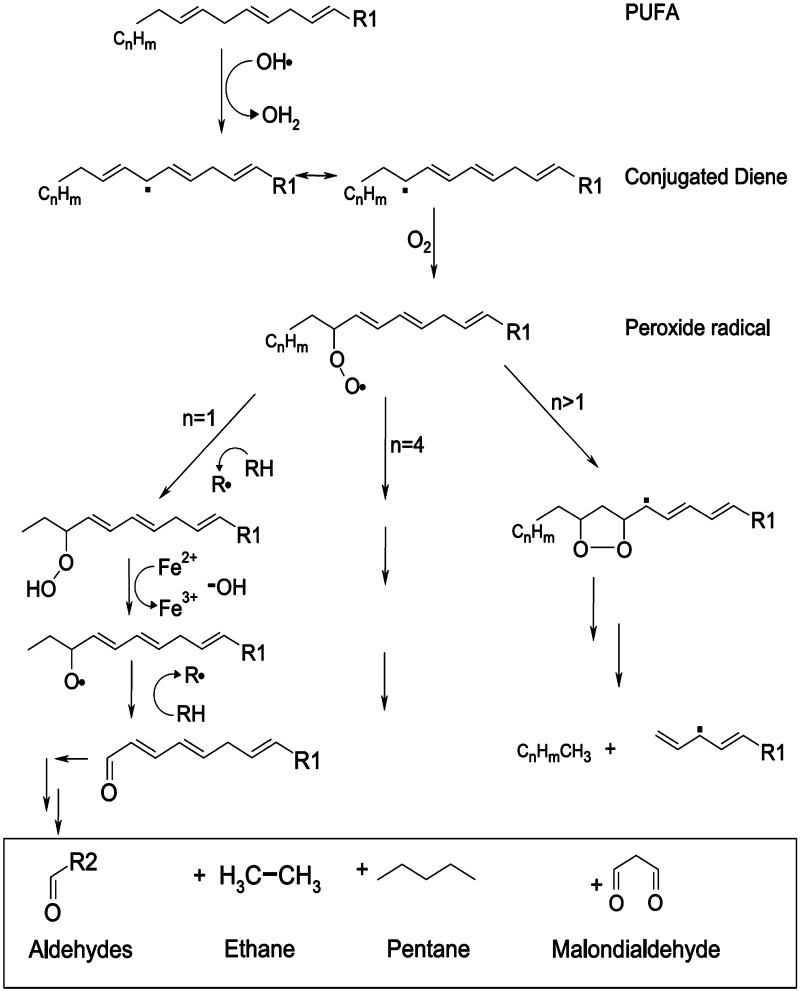
Possible reactions and their products in free-radical-mediated lipid peroxidation [2]

Saturated hydrocarbons, e.g., ethane and pentane, are produced from ω3 (e.g., linolenic acid) and ω6 fatty acids (e.g., linoleic and arachidonic acid), respectively (Fig. 2). These fatty acids have been described as the fundamental components of cell membranes [2, 6, 40, 43–45]. In vitro studies reveal that ethane and pentane originate when cell cultures are exposed to ROS. These aliphatic hydrocarbons are considered as in vitro and in vivo biomarkers of lipid peroxidation due to the close correlations between their exhalation and clinical conditions with high peroxidative activities, as suggested by various experimentations [46]. The levels of exhaled pentane and ethane are closely linked to other lipid peroxidation markers such as thiobarbituric acid reactive substances (e.g., malondialdehyde) [33]. Protein oxidation and colonic bacterial metabolism, despite being other important sources of hydrocarbons in the human body, have less potential in a hydrocarbon breath test for ethane and pentane [43]. Methylated hydrocarbons are also considered lipid peroxidation markers [2, 47, 48].

Several diseases, e.g., lung cancer [49], HIV [50], and inflammatory bowel disease [51], are also influenced by oxidative stress induced by ROS generation. Further study is required to conceptualize the biochemical process of the generation and physiological interpretation of these compounds [2, 52]. Such hydrocarbons are stable end products of lipid peroxidation [2, 6, 45]. They are less soluble in blood and are hence released into the breath quickly after their formation in tissues. Thus, exhaled ethane and n-pentane concentrations have potential in the monitoring of oxidative stress in the body [45, 53, 54].

Elevated levels of ethane have been reported for patients with asthma, chronic obstructive pulmonary diseases (COPD), and cystic fibrosis [55–57]. Pentane has also been observed at increased levels in asthma patients [40, 58]. The concentrations of ethane and pentane are flow-dependent in asthmatic subjects [40].

#### Unsaturated Hydrocarbons

**Fig. 3:**
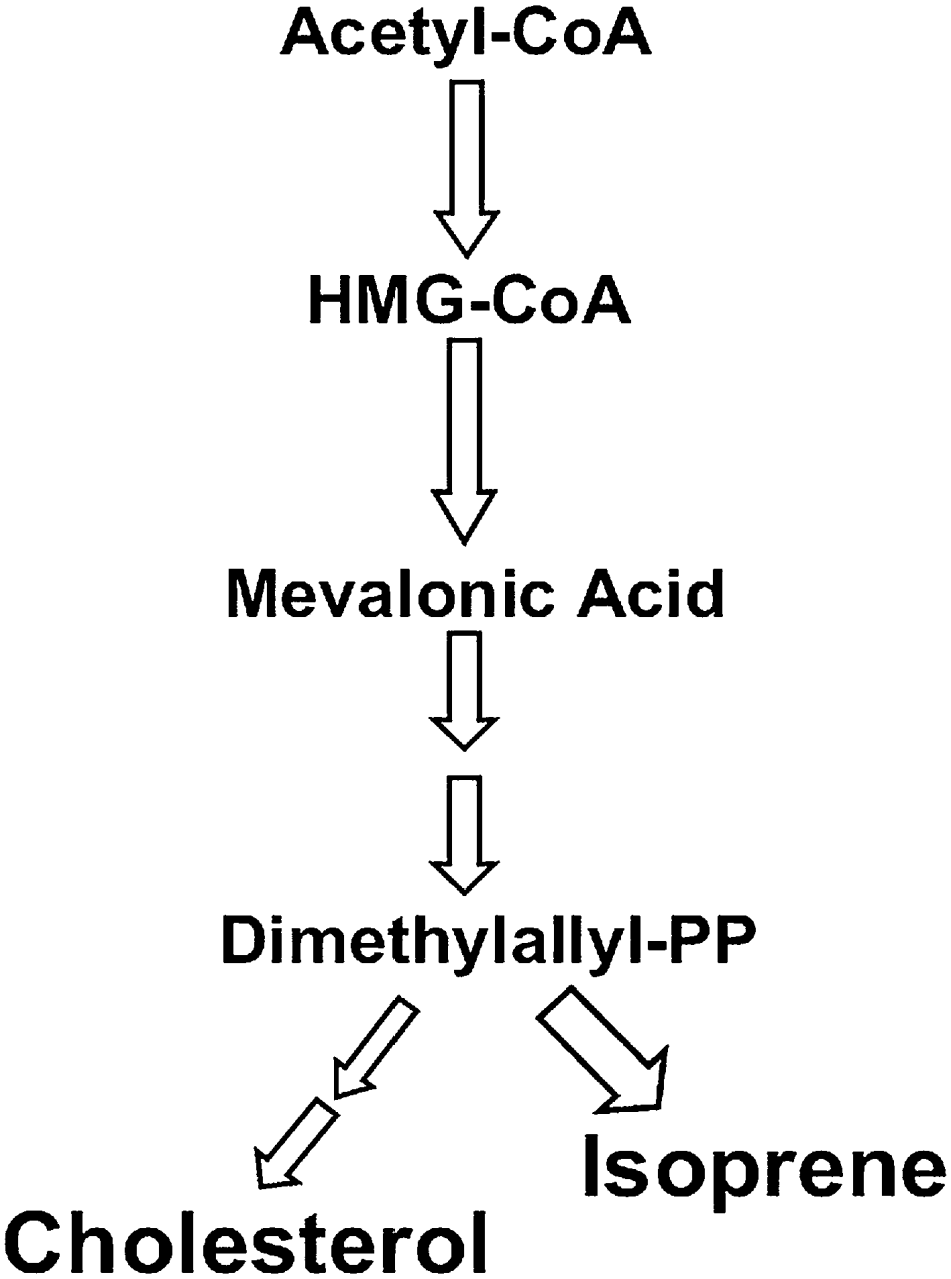
Metabolic pathway of cholesterol synthesis and isoprene generation [61]

Isoprene (2-methyl-1,3-butadiene) is the major hydrocarbon found in human exhaled breath [59, 60]. It is thought to be formed following the mevalonic acid (MVA) pathway (Fig. 3) of cholesterol biosynthesis [2, 40, 61–64]. The formation of mevalonate from acetic acid is a crucial chemical reaction in the cholesterol biosynthesis. The compound 3-hydroxy-3-methyl-glutaryl coenzyme A (HMGCoA) catalyzes the rate-limiting step of sterol synthesis. Next, mevalonate is converted in the cytosol to isopentenyl pyrophosphate, followed by isomerization to dimethylallyl pyrophosphate (DMPP) [65]. After passing through the formation of intermediate carbonium ion, DMPP is rapidly converted to isoprene via an acid-catalyzed elimination reaction [2, 66]. It has been reported that breath isoprene levels can be decreased in humans by administration of HMGCoA reductase inhibitors, which block the enzyme responsible for the production of MVA during cholesterol biosynthesis [2].

The 1-deoxy-d-xylulose-4-phosphate/2-C-methylerythritol 5-phosphate (DOXP/MEP) pathway is regarded as another pathway of isoprene generation in plants and most bacteria. In plants and bacteria, the enzyme isoprene synthase converts DMPP into isoprene [63]. The MVA pathway is mainly present in higher eukaryotes [67]. A small fraction of exhaled isoprene may be of bacterial origin [68]. However, the exact origins have not been found [66, 69]; further study on the origin of isoprene in the human body is needed.

Isoprene levels have been found to be decreased in patients with acute respiratory distress syndrome [70], cystic fibrosis [71], and asthma [40]. The concentration of isoprene in breath appears to be age-dependent. Nelson et al. [72] found that children show significantly low concentrations of breath isoprene. It has also been reported that isoprene levels in breath are increased during physical activity. Isoprene, stored in muscles, gets released during muscle contraction (i.e., it is exercise-induced) [64, 73]. The change in breath isoprene concentration can also be contributed to the alteration of diffusion capabilities of peripheral tissue [64]. Increased production of breath isoprene has been observed in human subjects and animals with chronic kidney disease (CKD). In CKD animals, elevation of isoprene levels is associated with a significant increase in serum cholesterol level [62]. Breath isoprene levels are also altered by physiological and pathophysiological states (including hemodialysis, general anesthesia, liver disease, and cancer [74, 75]).

### Sulfur-Containing Compounds

It has been reported that sulfur-containing compounds in the human body originate from the incomplete metabolism of methionine via the transamination pathway [2]. Such compounds, e.g., dimethyl sulfide, dimethyldisulfide, and ethyl mercaptane, manifest as a distinctive odor in cirrhotic patients’ breath [76–78]. Sulfur compounds are suggested as major markers of liver failure [2]. The breath of patients with hepatocellular failure may have a sweet, musty, or slightly fecal aroma, termed fetor hepaticus, which is mostly attributed to sulfur compounds. Dimethyl sulfide is regarded as an important metabolite for fetor hepaticus [79]. Impairment of liver function increases the level of these compounds, which have a characteristic smell, such as that of rotten cabbage [77, 80]. Bacteria and fungi related to lung infection (e.g., *Haemophilus influenza*, *Streptococcus pneumoniae*) also generate dimethyl sulfide [81]. Normally, human blood and breath shows very low (few parts per billion) concentrations of sulfur-containing compounds [5].

### Nitrogen-Containing Compounds

Identification and quantification of amines in human breath has been reported since 1977. Patients with chronic renal failure have been reported to exhibit characteristic uremic breath odor, which is termed ‘ammoniacal’, ‘fishy’, or ‘fetid’. Using GC, the presence of nitrogen-containing compounds (e.g., dimethylamine and trimethylamine at elevated concentration levels) contributing to the odor of uremic breath was established [82].

Ammonia is the major nitrogen-containing volatile biomarker. It originates in the body as a breakdown product of proteins. A substantial amount of these proteins comes from the bacterial degradation of proteins in the intestine. In the liver, ammonia is converted to urea and released in urine. Some of the ammonia is also released through the breath and the skin. Increased levels of ammonia have been observed in the blood when the liver fails to convert ammonia to urea. This may happen in subjects with cirrhosis or severe hepatitis [83].

Breath ammonia concentration is also influenced by cigarette smoking [84]. A relatively high concentration range [245–2935 ppb] of breath ammonia has been reported in normal healthy humans [83]. Such elevated levels may also be related to bacterial production in the oral cavity [5].

In the context of disease diagnosis using breath VOCs, several factors should be taken into consideration. The biology underlying both health and pathology is a complicated, non-linear, and dynamic process [85]. Human breath includes a complex composition. Food products, inhaled air composition, and volatiles that originate from normal and disease-related metabolic processes influence breath VOC content [23, 28, 86, 87]. For example, genomic and structural changes in the lung epithelium can alter the VOC concentration developed in normal physiological processes [88, 89]. Environmental sources [e.g., diet and inhaled (polluted) air] contribute a major part of exhaled breath VOCs [90]. For a breath test for disease identification, VOCs from systemic origin should be emphasized and exogenous VOCs should be excluded. However, exogenous breath VOCs may undergo physiochemical changes before exhalation [91]. Bacteria present in the lungs, gut, and oral cavity in animals and humans are potential VOC producers [92, 93]. Thus, VOCs of microbial origin in the human body cannot be overlooked and may be utilized in disease diagnosis.

A large number of VOCs have been found in human breath. Although many of them have been reported to be biomarkers of several diseases, their exact physiological interpretation, including their biochemical status both in diseased and healthy conditions of the subjects, needs further study. It should be noted that the concentration ranges of breath VOCs for diseased persons and normal healthy subjects are not similar. For example, some exhaled breath VOCs and their concentration ranges in normal healthy subjects and lung cancer patients are given in Table 1.

**Table 1:** Various breath VOCs and their concentrations in healthy normal subjects and lung cancer patients

Sl. no.	VOC	Concentration (range/median/mean)^a^ values (ppb)	
		Normal healthy subjects	Lung cancer patients
1	Isoprene [3, 10, 15, 16, 208]	12.71 [209] to 227 [210]	80–225.6 [208]
2	Acetone [3, 15, 16, 199, 211–213]	44 [199] to 2744 [83]	150–870 [131]
3	2-Pentanone [15, 199]	1.80–4.11 [199]	3.25–8.77 [199]
4	Ethylbenzene [3, 15, 16, 199, 208, 213, 214]	0.28 [208] to 18.38 [199]	1.45–3.16 [199]
5	Xylenes	0.54–1.43 [208]	1.1–2.76 [208]
6	Toluene [199, 208, 211–213]	1.45–37.21 [199]	–
7	Ethane [15, 215]	0.51–1.02 (non-smokers) [216]	–
8	Pentane	6.84–14.36 [199]	1.73 [199] to 28.3 [208]
9	Propane [212–214]	3.71–19.98 [212]	–
10	Ethanol [2, 15, 16, 211–213, 215]	27 [217] to 216.1 [218]	64–2160 [131]
11	Methanol [2, 16, 213]	33.05–216.1 [218]	63–110 [131]
12	Undecane [15, 16]	0–4.83 [219]	–
13	Nonanal [15, 16, 220, 221]	0.18 [220] to 2.44 [221]	0.8 [220] to 12.61 [221]
14	Benzene [3, 15, 16, 199, 208, 213, 215]	0.7 [208] to 14.97 [199]	1.29–3.82 [199]
15	Heptane [3, 15, 16, 208, 215]	0.13–0.39 [208]	0.04–0.86 [208]
16	2-Methylpentane [15, 16, 199, 208, 213, 215]	0.08 [208] to 10.80 [199]	0.93 [199] to 7.6 [208]
17	Butanal [199, 211, 215, 220, 221]	0.18 [220] to 72.2 [211]	0.48 [220] to 131.4 [211]
18	Acetonitrile [199, 211–213]	5.99–28.98 [199]	10.96–23.60 [199]
19	Butane [16, 211–213]	3.6–14.8 [211]	3.3–9.2 [211]
20	2-Propanol (isopropanol) [15, 16, 199, 213]	3.21 [199] to 94.1 [13]	84.2–340.7 [211]
21	3-Methylpentane [15, 16, 199, 212, 213]	1.05–8.76 [199]	0.94–8.87 [199]
22	Hexane [15, 16, 199, 211–213]	0.29–12.86 [212]	1.44–1.88 [199]
23	Styrene [16, 208, 213]	0.14–0.55 [208]	0.22–0.95 [208]
24	Octane [15, 16, 208, 213]	0.1–1.29 [208]	0.57–2.87 [208]
25	Decane [15, 16, 208, 213]	0.36–10.3 [208]	7.1–33.6 [208]
26	1,2,4-Trimethylbenzene [15, 16, 208]	0.12–0.28 [208]	0.24–0.56 [208]
27	Hexanal [15, 220]	0.18–0.35 [220]	0.68–1.47 [220]
28	Pentanal [15, 220]	0.11–0.37 [220]	0.32–1.08 [220]
29	Methanethiol [212]	1.83–2.87 [212]	–
30	Dimethyl sulfide [211, 212]	6.9–7.9 [211]	0–6.9 [211]
31	2-Methyl-1,3-butadiene	96.4 (median) [211]	132.7 (median) [211]
32	1-Butanol [222]	0.75 (median) [222]	–

^a^Data in other units (e.g., nmol/l, μg/l, etc.) are converted to equivalent ppb unit, considering breath temperature at 310° K and following the conversation formula applied in Ref. [212]

## Underlying Principle in Exhaled Breath Analysis

The underlying principle of breath analysis is based on the relatively rapid equilibrium between alveolar air and pulmonary capillary blood [94]. Following Henry’s law, it can be stated that the quantity of a given substance released through the lungs varies in proportion to its vapor pressure [95]. Due to the rapid gas exchange at the blood-gas interface in the lungs, the gaseous compounds present in expired breath are directly proportional to their blood concentrations. However, no specialized transport system has been reported for the excretion of lethal gaseous substances by the lungs; such substances seem to be eliminated by the process of simple diffusion [94, 95]. Also, the elimination of gaseous substances is inversely proportional to their absorption by the lungs [95]. This mechanism holds true for any gas or vapor with no special affinity for certain blood components. During inhalation, gas molecules diffuse from the alveolar space into the blood and become dissolved. The uptake of gas molecules by tissues utilizes a simple physical dissolution process, facilitating the partition of these molecules between the air and blood during absorption and between blood and other tissues during distribution.

Any chemical substance in the alveoli stays there sufficiently long to reach a state of equilibrium with the blood. However, due to continuous contact of the inspired with blood, the state of equilibrium occurs rapidly. The ratio of the concentration of the chemical in the blood to that in the gas phase is constant at this equilibrium, termed the blood-gas partition coefficient (*K*), and is unique for every gas [95].

Due to the simple mechanism described above, the VOCs in breath can be an excellent indicator of the levels of these compounds in blood [94]. However, the biomonitoring and interpretation of VOCs in breath also requires special attention to factors that can influence pulmonary excretion. Such factors include metabolism, breathing technique, diffusion–adsorption–desorption, ventilation–perfusion, temperature, blood composition, and time [96].

### Significance of VOC Analysis in Human Breath Analysis and Applications

Regarding clinical prospects, breath samples have application in the following major areas:

1. *Clinical diagnosis* Here, the aim is to analyze VOCs produced in the organism and released through expired or exhaled breath.
2. *Exposure analysis* Here, the aim is to obtain quick and accurate information concerning the levels of highly toxic exogenous VOCs that reach the blood stream.

Clinical diagnosis, being a noninvasive and quick approach, has achieved a scientific research interest in the field of medical science and technology [1, 2, 97–100]. Exposure assessment is mainly used for detecting noxious substances in indoor environments, which is outside of the scope of the present review.

The determination of VOCs in breath samples can provide clinically significant information for the diagnosis of diseases and biochemical processes involved. Elevated levels of ethane and pentane have been reported as markers of oxidative stress in subjects with heart transplant rejection [1, 2, 101], breast cancer, bronchial asthma [1, 101], asthma, COPD, obstructive sleep apnea, acute respiratory distress syndrome (ARDS), and mental and physical stress [2, 101]. Methylated alkanes have also been proposed as markers of oxidative stress [1, 101]. Increased concentrations of exhaled n-alkanes have been observed following reperfusion of cardiopulmonary bypass [102] and in subjects with abdominal ischemia [103], ischemic liver [104], myocardial infarction [105], and ischemic heart disease [106]. Sepsis, or systemic inflammatory response syndrome, patients exhale significantly more pentane compared to that exhaled by healthy subjects [70]. Although few studies have found propane and butane (resulting from protein oxidation and fecal flora) to be biomarkers of lipid peroxidation [2], a recent study [42] claimed that patients with schizophrenia and bipolar disorder exhibit significantly increased levels of breath ethane and butane. Reduced isoprene levels have been found in patients with chronic heart failure [107] and ARDS [108]. Clinical studies demonstrated that isoprene may serve as a marker of cellular damage and repair. Increased concentration of isoprene has been reported in breath after smoking [109]. Several studies found elevated isoprene breath concentrations during and after hemodialysis [110]. Relatively higher concentrations of sulfur-containing compounds have been reported in liver transplant patients [111] and patients with liver disease [112]. The expired breath of cirrhotic patients includes ethyl mercaptane, dimethyl sulfide, or dimethyl disulfide, which are sulfur-containing compounds. Impairment of liver function also increases the level of sulfur-containing compounds [2, 101]. *Helicobacter* (*H*.) *pylori* infection can be detected using the urea breath test [1, 101]. Elevated levels of acetone in the expired breath of diabetes mellitus patients have been reported [2, 113]. The VOCs of cystic fibrosis and breast, colon, and prostate cancer patients have been detected in some recent studies [3, 101]. It has also been reported that a combination of more than 20 breath VOCs, predominantly alkanes, alkane derivatives, o-toluidine, aniline, and benzene derivatives, along with altered lipid peroxidation activity [1, 101], are able to distinguish patients with lung cancer at a probability level of about 70 %. Elevated levels of ammonia have been observed in the expired breath of patients with uremia, end stage renal failure [83, 84], acute liver failure, and hepatic encephalopathy [114–116]. Breath ammonia is also influenced by infection by *H*. *pylori* [114], which disturbs cerebral function [115] and produces symptoms of Alzheimer’s disease [116].

The breath test was applied by Humphreys et al. [117] to the diagnosis of pneumonia associated with invasive mechanical ventilation. Similar studies on pneumonia diagnosis have been carried out by Hockstein et al. [118] and Hanson et al. [119] with satisfactory outcomes. In a study on 60 asthma patients (21 fixed asthma patients and 39 classic asthma patients) and 40 COPD patients, Fens et al. [120] discriminated fixed asthma patients from COPD patients and classic asthma patients, respectively. Remarkable results were also reported by Fens et al. [121] when they compared the exhaled VOCs from 20 asthma patients, 30 COPD patients, 20 non-smoker controls, and 20 smoker controls. Another research group [122] analyzed the exhaled air from 10 controls and 18 COPD patients. All the COPD patients and 8 of the 10 controls were reported to be correctly identified. Dragonieri et al. [123] discriminated 10 asthma patients from 10 healthy subjects. Similar results were obtained by Montuschi et al. [124], who recruited 27 asthma patients and 24 healthy subjects. The breath test has also been utilized to diagnose other respiratory diseases, including tuberculosis [125–127] and apnea [128–130]. Machado et al. [131] compared the exhaled breath VOCs of 14 lung cancer patients with 54 control patients. In another study, Dragonieri et al. [132] compared the VOCs present in the breaths of 10 lung cancer patients with those of 10 healthy controls and 10 COPD patients, and found significant differences between the groups. Natale et al. [133] and Chen et al. [134] also performed breath tests to characterize lung cancer patients.

From the above discussion, it is undeniable that the analysis of VOCs in exhaled breath has potential for clinical diagnosis. However, the breath test has some shortcomings. The advantages and limitations of the breath test are listed in Table 2.

**Table 2:** Advantages and limitations of exhaled breath test

Advantages	Limitations
Noninvasive Repeated measurements allowed Possibly portable Real-time monitoring possible Quick measurements possible Personalized medicine (breath print)	Extraneous parameters, e.g., diet, environment Lack of standardization in breath collection Lack of standardization in analytical techniques Wide variability in results Storage of samples Physician acceptance

## Analytical Techniques Applied in Exhaled Breath VOC Analysis

Several techniques have been tailored and employed to monitor and analyze the VOCs present in human exhaled breath. Technologies used in the breath test are described below.

It should be noted that there are some potential obstacles in breath gas analysis. These include low concentrations of exhaled breath VOCs, lack of proper sampling and measuring techniques, and high humidity of breath gas [10]. The majority of breath VOCs have nanomolar (10^−9^) and picomolar (10^−12^) concentration ranges and require highly sensitive devices and sophisticated analytical methods for their proper identification [13]. Modern sampling and analytical methods have solved these problems to some extent.

The analysis of VOCs in exhaled breath requires some advanced technology that may pacify the analysis process and provide reliable interpretation of the obtained data. In this regard, incorporating sensors and sensor arrays might be useful in the analysis of expired breath VOCs of diabetes patients.

### Gas Chromatography and Gas Chromatography–Mass Spectrometry

Commonly used methods for the analysis of exhaled breath VOCs are GC, GC–MS, and GC with tandem MS (GC–MS^2^) [4, 7, 61, 98]. In practice, GC/MS systems are often associated with other methods, e.g., solid-phase extraction (SPE) and solid-phase microextraction (SPME), for the analysis of breath VOCs [1]. In breath analysis, GC–MS employs the electron impact ionization technique. The unique fragmentation pattern of each analyte molecule is used for its identification, which is done using chromatographic retention data and mass spectral data using spectral libraries [98]. GC–MS has high reproducibility, high sensitivity, and robustness [135] and conveys potential information of biological mechanisms relevant to respective VOCs [136, 137].

Combined with a preconcentration system, these methods can be effectively applied to the off-line collection, separation, and identification of the majority of compounds (e.g., aliphatics, alcohols, aldehydes, ketones, amines, and halogenated compounds) in human breath [4, 7, 98]. GC and GC–MS have numerous applications in clinical studies [2, 138]. These systems are sufficiently sensitive to quantify breath gases of very low concentration (parts per billion/ppb/ppbv—parts per trillion/ppt/pptv) levels [139, 140].

However, despite their high sensitivity, GC–MS and related methods have poor portability and are time-consuming, costly, difficult to handle, and not suited for real-time and repeated measurements [10]. Therefore, GC–MS is used especially when the aim is to identify VOCs in pathophysiological discovery, even though it can also be clinically incorporated in larger hospitals when logistics allow samples to be directly analyzed.

### Proton-Transfer Reaction-Mass Spectrometry

Compared to GC–MS, proton-transfer reaction-mass spectrometry (PTR-MS) is more sensitive in detecting VOC concentrations, down to ppt [13] and ppb levels [7, 61, 141], quickly and accurately and does not require the time-consuming preconcentration step [13]. In PTR-MS, analytes are characterized as per their mass-to-charge (*m/z*) ratio only. PTR-MS includes an ion source and a drift tube (ion-transfer region) coupled to an MS detector. H_3_O^+^ ions generated in the ion source region are mixed with an air sample continuously flowing at the top of the drift tube and proton transfer takes place as the gas sample passes through the drift tube [142].

PTR-MS has been used for monitoring anesthetic agents [143], breath profiling [144], and studying the effects of hemodialysis [145]. This technique is also suited for online and multiple measurements. However, PTR-MS systems cannot differentiate between substances that have the same molecular mass [13]. Chemical identification of the analytes thus cannot be performed using this technique. For this purpose, other techniques should be used [7, 10].

### Selected Ion Flow Tube-Mass Spectrometry

Similar to PTR-MS, selected ion flow tube-mass spectrometry (SIFT-MS) shows quick performance in quantifying exhaled breath analytes [4, 7, 61]. SIFT-MS employs a chemical ionization technique using positive precursor ions (H_3_O^+^, NO^+^, or O_2_^+^) generated in an ion source. The ion–molecule reactions in SIFT-MS generate characteristic product ions corresponding to each trace gas species present in the sample. These product ions are mass-sorted and counted by a downstream detection system. SIFT-MS has been applied to polar substances (e.g., acetone, ammonia, acetaldehyde, and alcohols) and unsaturated hydrocarbons (e.g., isoprene) [98].

SIFT-MS is a potential real-time quantification tool for several breath gases [4, 61, 146–148]. It can detect several VOCs with concentrations at ppt/pptv and ppb/ppbv levels [7, 61, 98]. SIFT-MS outperforms GC–MS in terms of the identification and analysis of small molecules [149]. For a specified mass range, a large number of target compounds can be detected and monitored simultaneously.

### Sensor Arrays and Electronic Noses

Sensor technology has been utilized in several clinical tests (e.g., blood gas analysis) for many years [98]. Sensor systems are characterized by their high throughput, ease of use, and affordability [150]. With the aid of modern fabrication techniques, sensor arrays find their notable implementations in optimizing the conditions for clinical applications. One of the major sensor-based techniques in the clinical domain is the electronic nose (e-nose), which incorporates a series of non-selective gas sensors and a pattern-recognition algorithm [4, 150, 151]. The pattern of volatile compounds may represent not only an infecting organism or a disease state but also the metabolism of the host response, as well as other associated conditions [97, 152].

An e-nose, resembling the human (or animal) nose, can recognize the ‘smell-print’ to which it is trained [7, 61]. Utilizing nanosensor arrays, an e-nose detects patterns in complex mixtures of exhaled breath VOCs. Such patterns may serve as potential indicators of certain disease conditions [7, 97, 121, 131, 152] and can pacify the clinical diagnosis process. E-noses have been used for detecting pathogens present in bacterial cultures [1, 153] and determining halitosis [1], uremia [154], renal disease, airway inflammation [1, 155], cancer [1, 3, 13, 132, 156], malignant pleural mesothelioma [157], and pulmonary diseases [1, 120, 121, 127, 132].

### Ion-Mobility Spectrometry

Ion-mobility spectrometry (IMS) is a fast technique that can detect volatile compounds of very low concentrations (ppm–ppb) without any preconcentration [7, 98]. It is based on estimating the time taken for an ion to travel through a drift tube. Variations in the humidity of an exhaled breath sample may influence the ion-drift time [7]. IMS can separate and identify different substances under ambient air conditions. IMS can be coupled with GC [158] or MS [159] for unequivocal identification of substances.

IMS measures disease-specific combinations of VOCs, and characterizes the mixture using a pattern recognition algorithm to aid clinical diagnosis. Wide application of hand-held IMS units has been reported in military applications [160]. IMS has been used to identify some metabolites [161] and bacteria in human breath [162]. Unfortunately, IMS cannot identify unknown volatile compounds in a sample [161, 163].

### Optical Absorption

Optical absorption can be employed to identify a disease once a specific molecule associated with the disease has been recognized. This method is very selective and can be used to perform on-line real-time analysis of a specific compound at the ppb level [7]. Chemiluminescence analyzers employed for measuring fractional concentrations of exhaled nitric oxide (FeNO analyzers) work on the principle of optical absorption. Such instruments are characterized by their high cost, large size, and poor portability. However, low-cost portable instruments based on the electrochemical cell technique have been recently developed [164].

Table 3 gives a brief summary of the applications, advantages, and disadvantages of analytical methods used in breath testing.

**Table 3:** Characteristics of various analytical techniques used for breath test [7, 61, 98]

Analytical method	Typical compounds	Limit of detection	Advantages	Limitations
GC–MS	Sulfides, hydrocarbons, aldehydes	ppt–ppb ppt–ppb ppt–ppb	High selectivity and sensitivity	Large sampling time/requires standardization/requires preconcentration
PTR-MS	Aromatic compounds, isoprene	ppt ppt	Analysis is real-time	Small range of detectable compounds/compounds cannot be identified
SIFT-MS	Ethanol, ammonia	ppt–ppb ppt–ppb	Analysis is real-time/wide range of detection	Compounds cannot be identified
Sensor arrays/e-noses	Various VOCs	N/A	Analysis is real-time/potential for portability and miniaturization	Pattern recognition makes identification of compounds impossible
Ion mobility	Isoprene, acetone, ammonia	ppt–ppb ppt–ppb ppt–ppb	Vacuum systems are not required and ambient air can be used as a carrier gas	Not very useful for identifying unknown compounds in multi-component mixtures
Optical absorption	Ethane, carbon monoxide	ppt ppt	Analysis is real-time/potential for portability and miniaturization	Limited by available technology to meet sufficient specificity/selectivity required for practical use

## Diabetes Mellitus and Breath Test

### Diabetes

Diabetes mellitus, one of the major diseases, poses a great threat to human health and has become a universal epidemic [165–167]. According to the World Health Organization (WHO), approximately 350 million people globally have diabetes, which is projected to be the 7th leading cause of death in the year 2030 [166, 168, 169]. Diabetes also refers to a variable and complicated disease condition that can severely affect almost every organ in the human body. This disease may be considered as a combination of several diseases with versatile origins, various ages of onset, and multiple treatment requirements [167].

Diabetes manifests itself as an uncommon combination of dramatic symptoms and has a lethal outcome [170]. It is a common metabolic disorder characterized by chronic hyperglycemia that can be potentially life-threatening. Improper management of this deadly disease may lead to damage of the eyes, kidneys, heart, and nerves [166, 171].

### Classification of Diabetes

WHO classifies diabetes into type 1 diabetes (insulin-dependent diabetes mellitus) and type 2 diabetes (non-insulin-dependent diabetes mellitus). Type 1 diabetes stems from an absolute insulin deficiency. Usually, it is an autoimmune disease and can lead to the destruction of the insulin-secreting beta cells in the pancreas. However, the origin of the destruction of beta cells is unknown in some cases. Type 2 diabetes results from a relative insulin deficiency that may be associated with varying degrees of insulin action defects, known collectively as insulin resistance [167]. It spreads in the body much more slowly than does type 1 and does not make people sick as fast. When it victimizes people, it can appear to be formidable one [116, 117].

Diabetes mellitus can appear in forms other than type 1 and type 2. It can also originate from gene mutations, pancreatic damage, and excessive hormone production. Diabetes can also be induced by medications. For example, Niacin is used to lower triglyceride levels and raise HDL cholesterol levels. Niacin lowers insulin effectiveness and can cause an increase in blood glucose levels (BGLs) [172]. Gestational diabetes mellitus (GDM) is another form of diabetes [172, 173]. It can develop when a pregnant woman has limited beta cell capacity and cannot respond to the additional insulin demand. An oral glucose tolerance test is the general approach for GDM screening. A recent study used breath gas analysis using PTR-MS for screening GDM [173].

## Breath Analysis for Diabetes Mellitus and Available Techniques

The management of any disease requires its timely and regular diagnosis. The traditional method for diagnosing or monitoring diabetes is collecting blood samples from patients to check whether their BGL falls within the normal range. This method is accurate but painful, invasive, inconvenient, and impractical and may be expensive [12, 174–176].

In addition, the measurement of plasma insulin has clinical importance for monitoring the progression of type 2 diabetes, discriminating common states (where components of both type 1 and type 2 diabetes are present simultaneously), and assessing and screening prediabetic states [174, 176]. Also, monitoring insulin is helpful in investigating aspects of metabolism. Insulin regulates glucose disposal and also exerts a strong antilipolytic effect that is reduced significantly in patients with insulin resistance [174, 177]. However, tests for insulin concentration and sensitivity are very laborious. Tests for circulating lipids are important for diabetic patients as hyperlipidemia is a critical risk factor for heart disease. As lipids enhance ketone body formation, any change in their levels may also be related to changes in insulin or glucose metabolism [174, 178]. Detailed knowledge and information of the interplay of these metabolic variables and their pathways may give clinical professionals insight into their patients’ health and hygiene, and reliable noninvasive monitoring, which would improve both the diagnosis and treatment of diabetes.

Researchers have thus focused on noninvasive methods for screening diabetes and predicting BGL. Such methods include bioimpedance spectroscopy, fluorescence technology, and iontophoresis [179, 180]. However, these methods suffer from a lack of specificity and inaccuracy due to subject movement and sweating, skin irritation, etc. [165, 179, 180]. Despite the number of studies carried out, no FDA-approved commercial device for screening diabetes and predicting BGL exists [181].

An emerging and promising noninvasive approach for diagnosing diabetes is the breath test. Exhaled breath consists of a large number of various gaseous VOCs. These VOCs may be used as noninvasive biomarkers of a number of diseases, including diabetes. It has been reported that concentrations of several breath biomarkers in diabetics show significant differences from those for normal healthy subjects. Also, the concentrations of some biomarkers are correlated with the BGLs of diabetics. Thus, it is possible both to screen diabetes and predict BGLs by analyzing the exhaled breath VOCs or breath biomarkers of diabetes patients. For clinical applications, breath analysis is a noninvasive approach. Analysis of breath biomarkers and their concentrations has great potential for detecting a disease, monitoring its progression, or monitoring therapy [165, 182].

Several investigations have been carried out to study the breath biomarkers of diabetes. Acetone and other VOCs [165, 183–187] in breath have been shown either to have abnormal concentrations in diabetics compared to healthy controls or to correlate with the BGL. Compared to other noninvasive approaches for diabetes management, breath analysis is more advantageous and readily acceptable and it is easier to collect samples for it [174], making it an attractive and effective method for noninvasive diabetes screening and BGL prediction [174, 175].

## Physiological Aspects of Diabetes Mellitus Related to Breath Test

The diagnosis, screening, or monitoring of a disease using the breath test is not a simple task. Implementation of breath test for detecting a disease and accurate interpretation of the measured data not only require details of the pathophysiological background and systemic metabolism underlying the particular disease condition of the subject but also an elaborate understanding of the same under normal healthy conditions. Additionally, the methodology and technology used for such a test affect conclusions regarding the disease. Characterization, interpretation, quantification, and analysis of VOCs found in breath for a diseased subject are of great importance as those of for a healthy person for a significant statistical interpretation of the obtained data.

An accurate breath test for diabetes mellitus needs the proper realization of the changes of pathophysiological mechanisms and systemic metabolism induced by the disease. Such changes may be useful for the diagnosis of diabetes [174]. The following discussion highlights the changes in the lungs and systemic metabolism in diabetics.

### Lungs

It is believed that both the pulmonary vasculature and lung function are affected by diabetes, which deteriorates the gas exchange kinetics of several VOCs. This should be taken into consideration when incorporating VOCs in the breath test for diabetes. Further progress of the disease imparts severe pathological changes, which would require frequent recalibration or prevent specific VOCs from being used in the breath test. It should be noted here that although the underlying pathophysiological mechanisms are not fully understood, several studies have investigated such mechanisms in diabetics.

It has been reported that there is a correlation between diabetes (both types) and mild restrictive lung disease and micropathology-induced declined diffusion capacity. Such micropathology originates from non-enzymatic glycation and results in stiffening of the lung tissues [174, 188]. Thicker epithelial and endothelial capillary basal laminae have been found in diabetes patients after autopsies [174, 189]. Diabetic subjects also manifest elevated levels of plasma myeloperoxidase (which produces damaging oxidative species) and α-defensins (which promote atherosclerosis and lung injury) [155, 174, 190–192] and have increased risk of ROS production, pulmonary hypertension (type 2 patients), and enhanced pro-sclerotic and pro-fibrotic growth factors [174, 193, 194].

### Systemic Metabolism

The breath composition of diabetic subjects is changed due to the metabolic activities involved with the disease. Altered BGLs affect the breath VOC concentrations. Glucose may also indirectly affect other VOC levels. This can be exemplified by a variation in the rate of acetone formation due to hyperglycemia, induced by the associated suppressive effect of physiological compensatory hyperinsulinemia, which at its elevated stage characterizes type 2 diabetes and suppresses lipolysis [174, 177]. Exhaled breath isoprene also changes due to alteration in cholesterol biosynthesis [74, 174]. Thus, the variation in concentrations of ketones and other VOCs may be manifestations of the changing metabolism of glucose and insulin.

## Relating Breath VOCs to Diabetes Mellitus

Several studies have been carried out to investigate exhaled breath VOCs related to diabetes mellitus. Breath analysis of diabetic subjects can provide information about their BGLs. Exhaled acetone has been reported to be an important biomarker of diabetes [195]. Since the 1960s, a large number of studies have been conducted on the analysis of acetone in the expired breath of diabetics [195–197]. Breath acetone is linked to ketoacidosis [174, 198]. Elevated levels of acetone in the blood and breath of diabetics are a key cause of the fruity or sweet smell of the subjects’ expired breath [195]. Studies have also found a high correlation between BGL and the concentration of breath acetone [165, 185, 186, 195, 197, 198] in patients with diabetes. The exhaled breath acetone concentration has been reported to be in the range of 0.044 ppm [199] to 2.744 ppm [83] for normal healthy subjects, 2.2 ppm [200] to 21 ppm [165, 201] for type 1 diabetes patients, and 1.76 ppm [165, 183] to 9.4 ppm [200] for type 2 diabetes patients. The elevation of acetone level indicates either a deficit of insulin in cells or cells’ inability to effectively utilize available insulin [28]. Breath acetone can thus serve as a potential noninvasive biomarker of diabetes [28, 195].

However, the determination of breath acetone concentration alone may not optimize the diagnosis of diabetes as this concentration is also affected by the degree of insulin resistance, diurnal fluctuations, lipolytic activity, diet nutrient composition, gender [174], body exercise [28], and fasting status [28, 174]. Studies have shown the presence of other breath VOCs in the diabetics’ breath. A correlation between breath ethanol and BGL was reported by Galassetti et al. [195, 198]. Experiments on animals have also yielded interesting results. An elevated acetone level was found in obese mice after the application of a high-fat diet even after fasting [202]. Alteration in exhalation pattern and volatile metabolites was found during fasting and low-dose glucose treatment [203]. Hyperglycemia may influence medullary thyrotropin-releasing hormone stimulation of vagal outflow to the stomach [204].

Table 4 lists VOCs found in the exhaled breath of diabetes patients and normal healthy subjects. Few studies have reported the concentration ranges of breath VOCs, except for acetone, for diabetes mellitus patients.

**Table 4:** VOCs found in breath of diabetes patients and healthy normal subjects

Sl. no.	VOC	Type of diabetes	Applied method	Concentration (ppb) (range/median/mean)^a^	
				Normal healthy subjects	Diabetes mellitus patients
1	Acetone [165, 174, 182, 200, 201, 214, 223–226]	Type 1 Type 1 and 2 Type 2	Gas chromatography [214] Sensor-array-based method [165] Solid-phase micro extraction GC–MS analysis [223]	44 [199] to 2744 [83]	2200 [200] to 21000 [165, 201] (type 1) 1760 [165, 183] to 9400 [200] (type 2)
2	Ethylbenzene [174, 214]	Type 1	Gas chromatography [214]	0.28 [208] to 18.38 [199]	NA
3	Xylene [174, 223]	Type 2	Solid-phase micro extraction GC–MS analysis [223]	0.54–1.43 [208]	NA
4	Toluene [174, 223]	Type 2	Solid-phase micro extraction GC–MS analysis [223]	1.45–37.21 [199]	NA
5	Ethane [174, 227]	Type 1 and 2	GC–MS analysis [227]	0.51–1.02 [216]	NA
6	Pentane [174]	Type 1 Type 1 and 2	Gas chromatography [214] GC–MS analysis [233]	6.84–14.36 [199]	NA
7	Propane [174, 214]	Type 1	Gas chromatography [214]	3.71–19.98 [212]	NA
8	Isoprene [174]	Type 2	GC–MS analysis [212]	12.71 [209] to 227 [210]	NA
9	Ethanol [174, 214]	Type 1	Gas chromatography [214]	27 [217] to 216.1 [218]	NA
10	Methanol [174, 214]	Type 1	Gas chromatography [214]	33.05–216.1 [218]	NA
11	Isopropanol [223]	Type 2	Solid-phase micro extraction GC–MS analysis [223]	3.21 [199] to 94.1 [13]	NA
12	2,3,4-Trimethylhexane [223]	Type 2	Solid-phase micro extraction GC–MS analysis [223]	NA	NA
13	2,6,8-Trimethyldecane [223]	Type 2	Solid-phase micro extraction GC–MS analysis [223]	NA	NA
14	Tridecane [223]	Type 2	Solid-phase micro extraction GC–MS analysis [223]	NA	NA
15	Undecane [223]	Type 2	Solid-phase micro extraction GC–MS analysis [223]	0–4.83 [219]	NA

*NA* not available in the literature

^a^Data in other units (e.g., nmol/l, μg/l, etc.) are converted to equivalent ppb unit, considering breath temperature at 310° K and following the conversation formula applied in Ref. [212]

## Conclusion

This review paper reflects the research trends and developments of breath analysis in disease determination since 1966. The status of characteristic breath VOCs (mostly of systemic origin) at various physiological states was discussed. The advantages and limitations of breath tests and related analytical methods were highlighted. The application of breath VOC analysis to the diagnosis of diabetes mellitus was reviewed. 15 VOCs found in diabetics’ breath were listed, along with a list of 50 VOCs and their concentration ranges for normal healthy persons. The concentration ranges of a few breath VOCs found in disease conditions (e.g., lung cancer) were also highlighted.

Despite the potential of breath tests for the clinical diagnosis of several diseases, such tests have some drawbacks. The major challenge limiting the application of breath analysis in the clinical domain is the lack of standardization in both breath collection [4, 5, 205] and analytical approaches [4, 205, 206], which contribute to the wide variations of reported analytical results [5, 99, 182, 207]. Additionally, insight into the metabolic aspects and background physiology of breath VOCs and their exact contributions both to healthy and diseased states is required to justify their clinical importance from a diagnostic point of view. Breath tests are currently rarely used in clinical practice. However, it is expected that ongoing research and development in medical science and sensor-based analytical instrumentation in breathomics will establish breath analysis as a reliable approach for the detection and monitoring of various diseases.
